# Correlation between the Severity and Type of Acne Lesions with Serum Zinc Levels in Patients with Acne Vulgaris

**DOI:** 10.1155/2014/474108

**Published:** 2014-07-24

**Authors:** Majid Rostami Mogaddam, Nastaran Safavi Ardabili, Nasrollah Maleki, Maedeh Soflaee

**Affiliations:** ^1^Department of Dermatology, Imam Khomeini Hospital, Ardabil University of Medical Sciences, Ardabil 5618953141, Iran; ^2^Department of Midwifery, Islamic Azad University, Ardabil branch, Ardabil 5615731567, Iran; ^3^Department of Internal Medicine, Imam Khomeini Hospital, Ardabil University of Medical Sciences, Ardabil 5618953141, Iran

## Abstract

Acne vulgaris is the most common cutaneous disorder affecting adolescents and young adults. Some studies have reported an association between serum zinc levels and acne vulgaris. We aimed to evaluate the serum zinc level in patients with acne vulgaris and compare it with healthy controls. One hundred patients with acne vulgaris and 100 healthy controls were referred to our clinic. Acne severity was classified according to Global Acne Grading System (GAGS). Atomic absorption spectrophotometry was used to measure serum zinc levels. Mean serum level of zinc in acne patients and controls was 81.31 ± 17.63 *μ*g/dl and 82.63 ± 17.49 *μ*g/dl, respectively. Although the mean serum zinc level was lower in acne group, it was not statistically significant (*P* = 0.598). There was a correlation between serum zinc levels with severity and type of acne lesions. The results of our study suggest that zinc levels may be related to the severity and type of acne lesions in patients with acne vulgaris. Relative decrease of serum zinc level in acne patients suggests a role for zinc in the pathogenesis of acne vulgaris.

## 1. Introduction

Acne vulgaris is the most common skin disease, affecting nearly 80 percent of persons at some time between the ages of 11 and 30 years [1–5]. Estimates of the prevalence of acne vulgaris in adolescents range from 35 to over 90 percent [6]. It can persist for years and result in disfigurement and permanent scarring, and it can have serious adverse effects on psychosocial development, resulting in emotional problems, withdrawal from society, and depression [2]. Acne vulgaris is a disorder of the pilosebaceous follicles characterised by comedones, papules, pustules, inflamed nodules, and canalising and deep, inflamed, and sometimes purulent sacs [7].

Typically, acne vulgaris occurs on areas of the body with hormonally sensitive sebaceous glands, including the face, neck, chest, upper back, and upper arms. Acne has four main pathogenetic contributors: follicular hyperkeratinization, increased sebum production,* Propionibacterium acnes* (*P. acnes*) within the follicle, and inflammation. The earliest change in the pilosebaceous unit was initially thought to be follicular hyperkeratinization, which is associated with both increased proliferation and decreased desquamation of keratinocytes lining the follicular orifice [8]. Initiating factors for this inflammatory process are unknown.

A potential role for diet in acne is controversial [9–12]. A study of 47,355 women in the Nurses' Health Study that used retrospective data collection to determine diet during high school found an association between acne and intake of milk [13]. The authors suggested that natural hormonal components of milk or other bioactive molecules in milk could exacerbate acne [13]. Two subsequent large prospective cohort studies (one involving boys and the other involving girls) also reported an association between milk ingestion and acne [14, 15]. All three studies were questionnaire based, requiring subjects to recall their dietary intake and self-diagnose acne and disease severity. Diet, particularly dietary glycemic index, saturated fat, trans fat, milk, and fish may influence or aggravate acne development [9]. A case-control study of 205 patients with clinician-confirmed moderate to severe acne and 358 controls with mild or nonexistent acne also found a possible association between milk consumption (more than three portions per week) and moderate to severe acne [16]. Glycemic load diet and frequencies of milk and ice cream intake may have a direct relationship with acne vulgaris [17].

Acne pathogenesis has recently been linked to decreased nuclear forkhead box transcription factor O1 (FoxO1) levels and increased serine/threonine kinase mammalian target of rapamycin complex 1 (mTORC1) activity [18–20]. The cell's nutritional status is primarily sensed by FoxO1 and mTORC1. FoxO1 links nutrient availability to mTORC1-driven processes: increased protein and lipid synthesis, cell proliferation, cell differentiation including hyperproliferation of acroinfundibular keratinocytes, sebaceous gland hyperplasia, increased sebaceous lipogenesis, insulin resistance, and increased body mass index [20].

Data on favorable effects of dietary factors such as zinc, omega-3 fatty acids, antioxidants, vitamin A, and dietary fiber on acne vulgaris are limited [21]. This study was conducted to measure the serum zinc level in patients with acne vulgaris and compare it with healthy controls. Also, we aimed to evaluate the relationship between serum zinc levels and disease severity in acne vulgaris patients.

## 2. Methods and Materials

### 2.1. Patients and Controls

This study was approved by the Ethics Committee of the Ardabil University of Medical Sciences. This was a prospective cross-sectional study. Patients presenting with acne vulgaris to the dermatology out-patient clinic of Imam Khomeini Hospital in Ardabil, Iran, between June 2012 and June 2013 were recruited as the study subjects. A total of 100 patients with acne vulgaris and 100 healthy controls were included. The two groups were matched for age and sex. Each patient had a complete dermatological examination including global acne grading system (GAGS).

The inclusion criteria for patients with acne vulgaris were as follows: being over 15 years of age, being at least a primary school graduate, not taking any medication for any purposes, and willing to participate in the study. Patients were excluded if they had any disfiguring facial condition other than acne vulgaris, history of active malignancy, under immunosuppressive treatment, liver cirrhosis, renal failure, pregnancy, alcoholism, malabsorption disorders, physical disability, any neurological disorder, or other physical diseases which might cause psychological distress. Written informed consent was obtained from the participants.

### 2.2. Global Acne Grading System (GAGS)

The GAGS is a quantitative scoring system to assess acne severity. It was first developed by Doshi and colleagues in 1997 [14]. The total severity score is derived from summation of six regional subscores. Each is derived by multiplying the factor for each region (factor for forehead and each cheek is 2, for chin and nose is 1, and for chest and upper back is 3) by the most heavily weighted lesion within each region (1 for ≥ one comedone, 2 for ≥ one papule, 3 for ≥ one pustule, and 4 for ≥ one nodule). The regional factors were derived from consideration of surface area and distribution and density of pilosebaceous units. The severity was graded as mild if the score was 1–18, moderate with scores from 19 to 30, severe with scores from 31 to 38, and very severe if the score is more than 38 [14].

### 2.3. Measurement of Serum Zinc Level

A 3 mL intravenous blood sample from the elbow cavity was taken from eligible cases and controls, and then samples were collected by venipuncture in EDTA coated vacutainer (BD vacutainer, USA) and were transported to the laboratory maintaining cold chain. The blood samples were centrifuged immediately at 3000 rpm for 10 min, and plasma was immediately stored at −40°C until the time of analysis. Blood samples were withdrawn by zinc-free plastic syringes and placed in zinc-free centrifuge tubes. Atomic absorption spectrophotometry (Varian Spectra AA-10 Model) was used to measure serum zinc levels. The normal value of serum zinc level in adults was accepted as 70–140 micrograms per deciliter.

### 2.4. Statistical Analysis

The statistical analysis of the data was done using SPSS software (Version 19.SPSS Inc., United States). The comparison of the continuous variables was accomplished with Student's *t*-test, and, for the comparison of the categorical variables, the chi-square test was used. The Pearson correlation analysis was used in the evaluation of the correlation between scores of the different scales and other relevant variables. A *P* value of <0.05 was considered statistically significant. The results were expressed as means ± standard deviations.

## 3. Results

A total of 100 acne vulgaris patients and 100 healthy volunteers were enrolled in this study. Each group included 17 (17%) male subjects and 83 (83%) female subjects. The mean age of the patients was 22.64 ± 2.43 years with a range between 16 and 48 years, and the mean age of the controls was 23.27 ± 2.36 years with a range between 17 and 52 years. The two groups showed no statistically significant differences in age or sex. The mean duration of acne was 34.21 ± 21.11 months with a range between 6 and 120 months. In the acne group, 83 (83%) subjects were single and 17 (17%) subjects were married. The mean duration of education in this group was 10.48 ± 2.76 years. In the control group, 85 (85%) subjects were single and 15 (15%) subjects were married. The mean duration of education in this group was 11.28 ± 2.16 years. None of the above demographic variables showed any statistically significant differences between the acne group and the control group.

The severity of acne was mild in 64 patients (64%), moderate in 32 (32%), and severe in 4 (4%).  Table 1 shows the distribution of acne according to location and type of lesion. Serum zinc levels in controls ranged from 11 to 114 micrograms per deciliter with a mean value of 82.63 ± 17.49 micrograms per deciliter. Serum zinc levels in acne patients ranged from 42 to 113 micrograms per deciliter with a mean value of 81.31 ± 17.63 micrograms per deciliter (Table 2). The serum zinc level was low in 23% of acne patients while 19% of control group subjects had zinc levels lower than normal. ANOVA test results demonstrated no significant difference in means of serum zinc between acne patients and healthy subjects (*P* value = 0.598).

Table 3 shows the mean serum level of zinc in relation to severity of the disease based on the paired *t*-test analysis. The serum zinc levels were lower in patients with moderate to severe acne compared with patients with mild acne. There was a correlation between serum zinc level and severity of acne, and the results were statistically significant (*P* value = 0.047). The serum zinc level and duration of acne were assessed, but the results were not significant (*P* value = 0.690).

Table 4 shows the relationship between serum zinc level and type of acne lesions based on the Pearson correlation test. There was a correlation between serum zinc level and type of acne lesions at the following locations and the results were statistically significant: (1) comedones on the left cheek (*P* value = 0.049), (2) papules on the forehead (*P* value = 0.039), (3) papules on the chest and upper back (*P* value = 0.016), (4) pustules on the right cheek (*P* value = 0.011), (5) pustules on the chin (*P* value = 0.008), and (6) pustules on the chest and upper back (*P* value = 0.006).

## 4. Discussion

Zinc is an essential trace element that is necessary for growth and development at all stages of life [22]. Zinc has been recognized as a distinct element since 1509 but was not identified as an essential mineral until the 1900s. In 1961, a link was established between zinc deficiency, endemic hypogonadism, and dwarfism in rural Iran [23]. Zinc plays a key role in physical growth and development, functioning of immune system, reproductive health, sensory function, and neurobehavioural development. Zinc might play an important role in the development of alterations in keratinocytes with aging [24, 25].

It has been estimated that around 33% of the world's human population has diets deficient in Zinc, but this ranges between 4 and 73% in different countries [26]. Mild zinc deficiency is associated with depressed immunity, impaired taste and smell, onset of night blindness, and decreased spermatogenesis. Severe zinc deficiency is characterized by severely depressed immune function, frequent infections, bullous pustular dermatitis, diarrhea, and alopecia [27]. Zinc and vitamin A are essential for normal epithelial development. A decreased serum zinc level could also lead to increased androgenic production, which influences the activity of sebaceous glands.

Treatment with zinc induces a significant increase in the expression of all the markers involved in innate immunity [28]. Recent studies have shown that Toll-like receptor- (TLR-) 2 expression, a receptor of the innate immune system, was increased in acne lesions and could play an essential role in acne-linked inflammation. Adapalene can modulate the epidermal immune system by increasing the CD1d expression and by decreasing the IL-10 expression by keratinocytes [29]. Inhibition of TLR2 surface expression by keratinocytes could be one of the anti-inflammatory mechanisms of zinc salts in acne [30]. Zinc requirements increase during pregnancy, mainly because of its utilisation during embryogenesis and fetal development, and use of zinc salts in pregnant women is beneficial in those with zinc deficiency but that has no harmful effects in those without zinc deficiency [31].

*Propionibacterium acnes* (*P. acnes*) is an anaerobic, Gram-positive skin microbe that resides in pilosebaceous follicles of the skin and is also found in the conjunctiva, oral cavity, intestinal tract, and external ear canal [32, 33]. Three major genetic divisions, known as types I, II, and III, are currently recognized. All of these types can be isolated from normal human skin, but only type IA is considered as the “acne-specific” subtype [34].* P. acnes* is accepted as a commensal bacterium and in some cases has been shown to play a protective role against invading pathogenic colonization [35]. This organism is considered to play an important role in the development of acne vulgaris. Zinc inhibits polymorphonuclear cell chemotaxis, inhibits the growth of* P. acnes*, and activates natural killer (NK) cells and the phagocytic capacity of granulocytes. Its anti-inflammatory activity in acne could also be related to a decrease in tumor necrosis factor- (TNF-) *α* and IL-6 production and modulation of the expression of integrins, mainly intracellular adhesion molecule- (ICAM-) 1 and leucocyte function associated antigen- (LFA-) 3 [21, 30].

Some investigators have reported an association between low serum zinc levels and acne vulgaris, while others have not found the same. In 1997, Michaelsson et al. [36] studied zinc levels in the serum, epidermis, and dermis of 73 patients with inflammatory acne. The dermal and epidermal zinc levels of male patients were significantly lower than the control group, while the serum zinc levels were similar. There was also no association between the serum levels and epidermal and dermal zinc levels [36]. In another study conducted by Michaelsson et al. [37], the serum zinc levels and retinol-binding protein (RBP) were determined in 173 patients with acne and compared with those of a control group. The RBP is a specific transport protein and its level in plasma reflects the amount of vitamin A available to the tissues. Zinc is essential for RBP synthesis and secretion in the liver. Patients with severe acne were found to have lower levels of RBP than either patients with mild acne or healthy subjects of the same age. In the case of males with severe acne, the mean serum zinc level was significantly lower than that of the control group. They suggested that the observed condition of low levels of zinc and vitamin A in the serum of patients with severe acne may provide a rationale for the clinically good effect of oral zinc treatment [37].

Amer et al. [38] have compared the serum zinc levels in 50 patients with acne vulgaris and 38 control subjects and found statistically significantly lower zinc levels in advanced grades of acne patients compared to the control group. The zinc levels in slight grades of female acne patients were also lower than control females and the levels in the affected males were lower than the control males [38]. Ozuguz et al. [39] evaluated serum vitamins A and E and zinc levels in 94 acne patients and 56 age- and sex-matched healthy volunteers as control group. All patients were assessed according to GAGS and grouped as mild, moderate, severe, and very severe. There was a negative correlation between acne severity and vitamin E and zinc levels. They offered supportive dietary measures with foods rich in vitamins A and E and zinc in the acne prophylaxis and treatment and stated that supportive treatment with these vitamins and zinc in severe acne may lead to satisfactory results [39]. These findings are inconsistent with results of our study.

In our study, there was no significant difference in serum zinc levels between acne patients and healthy subjects. There was a significant correlation between serum zinc levels with severity and type of acne lesions. Cochran et al. [40] evaluated the efficacy of topical zinc therapy in 30 patients with mild to moderate acne vulgaris. Over a 12-week period, no difference was noted between placebo- and zinc-treated participants in regard to either the number or the type of acne lesions. Zinc serum levels were not significantly elevated between the two regimens before, during, or after treatment. They suggested that topical zinc therapy alone is not of significant benefit in the treatment of acne vulgaris [40]. El Saaiee et al. [41] in 1983 studied serum copper, iron, and zinc in cases of acne vulgaris. The results revealed changes in the copper and iron content of the sera, although they were statistically not significant, and the serum zinc level showed no changes compared to the control group [41]. These findings are consistent with results of our study.

## 5. Conclusion

The results of our study suggest that lower serum zinc levels may be related to the severity and type of acne lesions in some patients with acne vulgaris. Relative decrease of serum zinc level in acne patients questions the role of zinc in the pathogenesis of acne vulgaris, and there is a need for further studies. The fact that the zinc levels decreased in severe acne suggests that there is a consumption of the zinc in the inflammatory process, rather like the impact of inflammation on vitamin C. It is worth noting that milk, while likely worsening acne, tends also to increase zinc levels. Thus, stopping milk might lower zinc levels and at the same time improve acne. This suggests the action of inflammation locally will consume the zinc locally and lower the serum level.

## Figures and Tables

**Table 1:** The frequency of acne group according to location and type of lesion.

Type of lesion	Papules	Pustules	Nodules	Comedones
Location of acne				
Forehead	63	64	4	15
Right cheek	87	83	9	12
Left cheek	88	91	12	19
Nose	65	60	14	9
Chin	57	61	17	11
Chest and upper back	48	50	5	4

**Table 2:** The mean serum zinc level in acne patients and controls.

Study subjects	Serum zinc (*µ*g/dL)		*P* value
	Range	Mean ± SD	
Acne group (*n* = 100)	42–113	81.31 ± 17.63	0.598
Control group (*n* = 100)	11–114	82.63 ± 17.49	

**Table 3:** The relationship between zinc levels and severity of acne in patients.

Acne severity	Number of patients (*n* = 100)	Serum zinc level, *µ*g/dL (mean ± SD)	*P* value
Mild	64	83.97 ± 17.32	0.047
Moderate	32	78.68 ± 18.12	
Severe	4	74.66 ± 15.26	

**Table 4:** Relationship between serum zinc level and type of acne lesions.

Type and location of acne lesions	Standard deviation	*r*	*P* value
Comedones on the left cheek	2.15	0.23	0.049
Papules on the forehead	2.52	−0.26	0.039
Papules on the chest and upper back	5.36	−0.35	0.016
Pustules on the right cheek	2.59	−0.28	0.011
Pustules on the chin	2.23	−0.34	0.008
Pustules on the chest and upper back	10.32	−0.39	0.006
